# Analysis of time-of-flight small-angle neutron scattering data on mesoscopic crystals such as magnetic vortex lattices^1^

**DOI:** 10.1107/S1600576722008226

**Published:** 2022-10-01

**Authors:** Emma Campillo, Maciej Bartkowiak, Oleksandr Prokhnenko, Peter Smeibidl, Edward M. Forgan, Elizabeth Blackburn

**Affiliations:** a Division of Synchrotron Radiation Research, Lund University, SE-22100 Lund, Sweden; b Helmholtz-Zentrum Berlin für Materialien und Energie, Hahn-Meitner-Platz 1, D-14109 Berlin, Germany; c School of Physics and Astronomy, University of Birmingham, Birmingham B15 2TT, United Kingdom; Technical University of Munich, Germany

**Keywords:** vortex lattices, superconductivity, time-of-flight neutron diffraction, small-angle neutron scattering, SANS

## Abstract

Bragg diffracted intensities and **q** values for crystalline structures with long repeat distances may be obtained by small-angle neutron scattering (SANS) investigations. An account is given of the methods, advantages and disadvantages of obtaining such data by the multichromatic time-of-flight method, compared with the more traditional quasi-monochromatic SANS method. This is illustrated with data obtained from high-magnetic-field measurements on magnetic vortex line lattices in superconductors on the former HFM/EXED instrument at Helmholtz-Zentrum Berlin. The methods have application to other mesoscopic crystalline structures investigated by SANS instruments at pulsed sources.

## Abstract 2

A method is presented for extracting quantitative information on superconducting vortex lattices and other mesoscopic structures from time-of-flight small-angle neutron scattering.

## 1. Introduction

Small-angle neutron scattering (SANS) is a very apt technique for measuring large-scale crystalline structures having repeat distances in the tens to thousands of ångströms. Flux line, or vortex, lattices (VLs) in superconductors are a typical application (Eskildsen *et al.*, 2011 ▸), but others include skyrmion lattices (Mühlbauer *et al.*, 2009 ▸) and other long-period helical magnetic structures or mesophases formed by nanoparticles in colloidal suspensions (Mühlbauer *et al.*, 2019 ▸). Taking the case of Bragg diffraction by vortex lattices, the parameters obtained are the VL structure and spacing and the VL form factors as a function of applied magnetic field and temperature. These data may be analysed in terms of the magnetic penetration depth and the coherence length of the superconductor. Information about the superconducting energy gap and the nature of the pairing may also be inferred. Traditionally, such measurements have been performed on quasi-monochromatic (Δλ/λ ≃ 10%) SANS instruments, and there has been little published use of the multichromatic time-of-flight (TOF) SANS technique for such measurements (Pautrat *et al.*, 2012 ▸; Bannenberg *et al.*, 2018 ▸; Li *et al.*, 2019 ▸; Campillo, Bartkowiak *et al.*, 2022 ▸).

Many years ago, the Christen formula (Christen *et al.*, 1977 ▸) was derived for the quasi-monochromatic case to relate the integrated intensity of a VL Bragg peak at wavevector **q** [*q* = (4π/λ)sinθ, where θ is half the scattering angle and λ is the wavelength of the incident radiation] to the magnetic form factor (the spatial Fourier component of the VL magnetic field at that **q**). Here, we demonstrate how to obtain the same information using TOF SANS. With the increasing availability of pulsed sources, such as the ISIS Neutron and Muon Source (UK), the Spallation Neutron Source (USA), J-PARC (Japan) and soon the European Spallation Source (Sweden), and the reduction in quasi-monochromatic instruments due to the closure of reactor sources, it is important to establish how VL studies may be carried out quantitatively on TOF SANS instruments. The information about VLs is obtained from the integrated intensity, position and **q** widths of the diffraction spots. The analysis described here may equally be used to obtain crystallographic information about other mesoscopic structures, for example the skyrmions studied by Bannenberg *et al.* (2018 ▸) and Li *et al.* (2019 ▸).

We have carried out several experimental studies (Campillo, Bartkowiak *et al.*, 2022 ▸; Campillo, Soda *et al.*, 2022 ▸; Cameron *et al.*, 2022 ▸) on VLs using the HFM/EXED neutron beamline (no longer in service) at the Helmholtz Zentrum Berlin (Prokhnenko *et al.*, 2015 ▸, 2017 ▸; Smeibidl *et al.*, 2016 ▸). This instrument was a multi-purpose instrument that operated exclusively in TOF mode and had a SANS option. In this paper, we primarily draw on examples from a sample of 98% detwinned YBa_2_Cu_3_O_7_ (Campillo, Bartkowiak *et al.*, 2022 ▸) to illustrate our analysis methods. We have also used these methods for high-field VL studies on twinned Y_0.85_Ca_0.15_Ba_2_Cu_3_O_7_ (Cameron *et al.*, 2022 ▸) crystals and Ba_1−*x*_K_*x*_Fe_2_As_2_ (Campillo, Soda *et al.*, 2022 ▸). In all three cases, we had results obtained from quasi-monochromatic SANS to compare with. The data reduction on HFM/EXED and subsequent analysis were carried out using the software package *Mantid* (Arnold *et al.*, 2014 ▸).

## 2. Bragg diffraction in TOF SANS

To generate a VL, a magnetic field must be applied to the superconductor; the flux lines then lie parallel to this magnetic field (with vortices of supercurrent around them) and the reciprocal-lattice vectors of the VL are therefore perpendicular to this field. If the magnetic field is aligned nearly parallel to the neutron beam, all of the Bragg reflections from the VL will be experimentally accessible in the SANS geometry.

The diffraction condition for a Bragg peak is set by the Bragg equation. In most VL SANS studies using a quasi-monochromatic neutron wavelength, the diffraction condition is met by altering the angle of the sample and magnet together with respect to the incoming neutron beam, and so the measurement is typically referred to as a rocking curve through the Bragg condition. If the relevant diffraction spot is perpendicular to the rotation axis (*e.g.* a horizontal spot with a vertical rotation axis) then the rocking angle ω moves **q** perpendicular to the Ewald sphere, the rocking curve will be centred on the Bragg angle θ_B_, and the intensity integrated over ω and the rocking curve width can be extracted. In the more general case, when the spot is at an angle β to the perpendicular, the rocking will be centred on θ_B_/cosβ; it moves **q** more slowly through the Ewald sphere and the angular width has to be corrected by the ‘Lorentz factor’ cosβ. The width is often reported in terms of angular width but more properly corresponds to a width in the *q*_*z*_ direction (**z** is parallel to the applied field). In the rest of this paper we will use ω to refer to the special case where the rotation axis is perpendicular to the spot.

**Figure 1:**
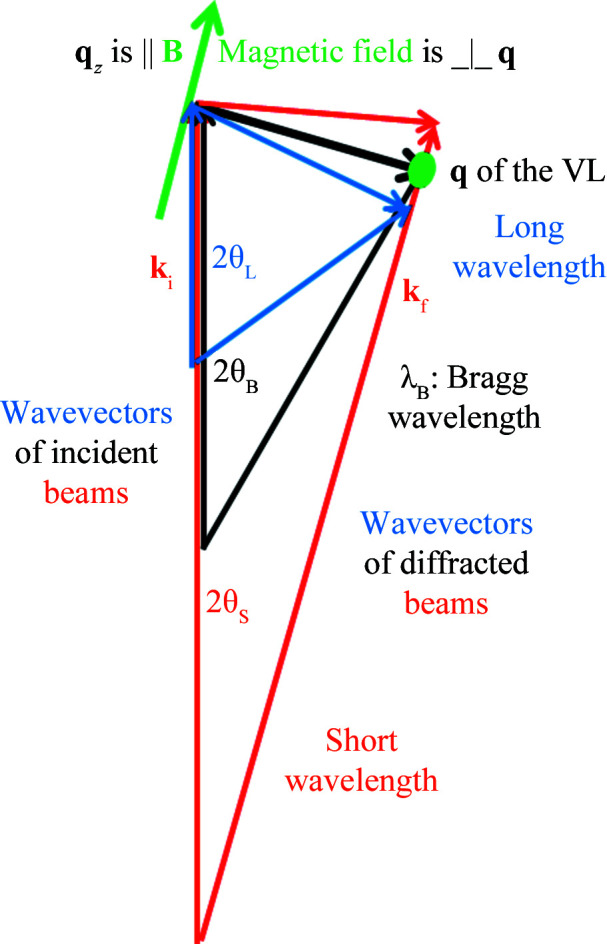
A schematic demonstration of how the TOF technique at one magnet rotation angle gives data equivalent to those obtained over a range of rocking angles in the single-wavelength technique. The spread of wavelengths in the incident beam is represented by three wavevectors of different lengths. The incoming and outgoing wavevectors **k**_i_ and **k**_f_ are labelled for one of these wavelengths. The magnetic field and sample are rotated together away from the incident beam direction by an angle which has been exaggerated for clarity. The rotation angle is chosen so that a VL diffraction spot satisfies the Bragg condition at a wavelength λ_B_ in the middle of the incoming wavelength distribution. Thus elastic scattering by 2θ_B_ (also exaggerated for clarity) corresponds to a VL reciprocal-lattice vector **q**, which is perpendicular to the magnetic field. Imperfections in the VL give a finite size to its diffraction spot, represented by the green ellipse, and this gives rise to different correlation lengths parallel and perpendicular to the field. For SANS measurements, the range of wavelengths or the rocking curve give information almost exactly along *q*_*z*_, the component of wavevector parallel to the field.

In the time-of-flight (TOF) case, at a given value of the sample and magnet rotation angle α a range of wavelengths are scattered, and so at each α a range of *q*_*z*_ values are sampled. Elastic scattering at the appropriate angles by shorter and longer wavelengths therefore corresponds to measuring the signal at different rocking angles in the case of quasi-monochromatic SANS ω (Pautrat *et al.*, 2012 ▸). Alternatively, as illustrated schematically in Fig. 1 ▸, the neutron wavelength distribution at fixed magnet rotation angle can be regarded as giving the signal over a range of *q*_*z*_. Depending on the range of Bragg angles and the width of the VL rocking curve, it may be necessary to measure in TOF mode at two or three different α values to give complete coverage of the rocking curve.

**Figure 2:**
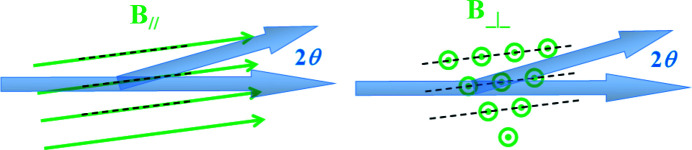
Illustrations of the two possible VL measurement geometries: (left) field nearly parallel to the incident beam and (right) field perpendicular. The relevant Bragg planes are shown as dashed lines

It is also possible to measure the VL with the field perpendicular to the beam (Laver *et al.*, 2008 ▸). In this case the sample has to be rotated so that one of the VL Bragg planes (which are typically 60° apart in this geometry) is at a small angle to the incoming beam (see Fig. 2 ▸). In this arrangement, the **B** direction marked in Fig. 1 ▸ corresponds to this Bragg plane and the field direction is perpendicular to the page. This means that the rocking curve or *q*_*z*_ width gives information about the orientation of the VL structure *around* the field direction rather than along **B**.

## 3. VL form factor

The VL form factor is the magnitude of a Fourier component of the spatial variation of the magnetic field within the VL. For non-TOF SANS at a neutron wavelength λ, the form factor *F*(**q**) for a diffraction spot with wavevector **q** is related to the integrated intensity *I*(**q**) by the following relationship (Christen *et al.*, 1977 ▸): 

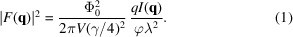

Here, Φ_0_ (= *h*/2*e*, with *h* the Planck constant and *e* the electron charge) is the magnetic flux quantum, *V* is the illuminated sample volume, γ (= 1.91) is the neutron magnetic moment in nuclear magnetons and φ is the neutron flux (neutrons m^−2^ s^−1^) illuminating the sample. The integrated intensity is defined as

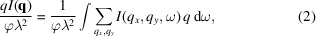

where ω is the rocking angle through the diffraction spot. (The angle is measured in radians, around an axis perpendicular to the scattering plane.) *I*(*q*_*x*_, *q*_*y*_, ω) is the intensity detected at rocking angle ω. This is then summed over the area of the detector containing the diffraction spot, and the integral represents the area under the graph of summed intensity versus *q*ω.

The equivalent equation in TOF mode is obtained by considering the illuminating beam as a distribution φ(λ) over the neutron wavelengths, represented by a histogram with bins of width Δλ centred on wavelengths λ_*j*_ (and corresponding wavevectors *k*_*j*_) having neutron flux φ_*j*_ = φ(λ_*j*_)Δλ. This will give rise to a scattered intensity *I*_*j*_ = *I*(*q*_*x*_, *q*_*y*_, *q*_*z*_, λ_*j*_)Δλ, with φ_*j*_ appearing in equation (1) and *I*_*j*_ inside the integral in equation (2). Note that Δλ cancels in the ratio *I*_*j*_/φ_*j*_, as indeed it does in non-TOF SANS where a single wavelength band is used. However, for a TOF measurement the VL is at a fixed angle to the neutron beam, so a rocking curve measurement is not performed. Instead, as shown in Fig. 1 ▸, the distribution of wavelengths corresponds to a range of values of the Bragg angle θ_B_, and hence plays the part of the rocking curve. If the range of wavelengths is not broad enough to cover the whole rocking curve width for a VL, several measurements at different magnet rotation angles may be used; the following analysis applies to each one of these measurements.

**Figure 3:**
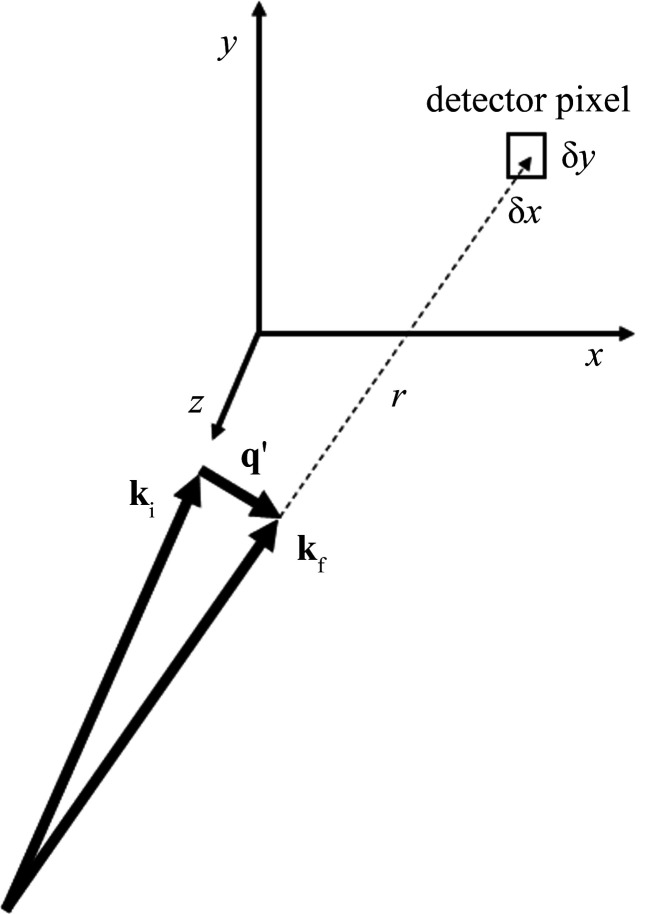
The geometry of the scattering. The incoming neutron beam at a particular wavelength is represented by **k**_i_ and a scattered neutron at the same wavelength with wavevector **k**_f_ arrives in a δ*x*δ*y* detector pixel centred (*x*, *y*) from the origin, after travelling a distance *r* to the pixel.

To do the TOF equivalent of ‘integrating over the rocking curve’, we have to determine how detector counts translate into **q** space. We make use of the geometry represented in Fig. 3 ▸, where we define the ‘laboratory’ frame, which has the *z* direction pointing towards the incoming beam.

In the laboratory frame, the scattered **q**′ = **k**_f_ − **k**_i_. If the magnitude of the neutron wavevectors is *k*_*j*_, then we may write the components of **q**′ as

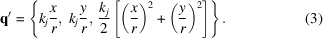

This is **q**′ in the laboratory frame; we wish to have **q** in the sample/magnet frame, which is rotated from the laboratory frame by a small angle α about the vertical *y* axis. If the diffraction spot is perpendicular to the magnet rotation axis then α is equivalent to ω, and α = ω = θ_B_ brings the spot into the diffraction condition. Otherwise α > θ_B_ is required. In our experiments the magnet rotation axis was vertical and the spots were at ∼45° to the horizontal, so α ≃ θ_B_/cos45°. In the sample frame, the VL diffraction spots will lie at *q*_*z*_ = 0, perpendicular to the field direction. For small rotation angles and small *q*_*z*_ we have, to a good approximation,

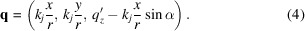

Differentiating equation (4) shows that the relationship between pixel space and **q** space in the *xy* plane is

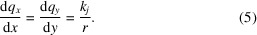




At this point, one has to make choices about how to treat the experimental information regarding wavelength and counts and how they are recorded. In our work here we have used *Mantid* as implemented at EXED, where we must work in **q** space, and so the rest of our treatment builds on that (see Section 5). However, different choices could be made, for instance in the user-friendly *GRASP* software (Dewhurst, 2003 ▸) work can be done using the appropriate TOF bins associated with each detector pixel.

Of immediate relevance for our discussion of the expression for the form factor is the fact that, when the detector data are assembled by *Mantid* into pixels of **q** space, there may be counts from several detector pixels added into one **q** pixel, and these counts will be normalized by the main beam intensity, summed for the same number of pixels. To calculate the intensity summed over the detector pixels, we have to account for this. If the size in the *xy* plane of a **q** pixel is Δ*q*_*x*_Δ*q*_*y*_ then, using equation (5), the area of the detector contributing to this pixel is Δ*x*Δ*y*, given by 

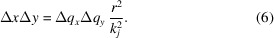

Hence the number *N* of detector pixels (of real-space size δ*x*δ*y* as in Fig. 3 ▸) included in this **q** pixel is

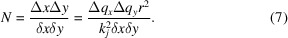

This factor has to be included in a sum over **q** space to convert it into a sum over the detector. We note that the 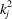 in the denominator cancels the 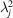 in the Christen formula for the whole of the wavelength distribution. The Christen formula applies when summing over a real-space detector; when summing over **q** space the wavelength dependence in the Christen formula disappears. Finally, *q*dω translates into d*q*_*z*_, so we come to the TOF version of the formula for the form factor,

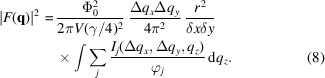

The area Δ*q*_*x*_Δ*q*_*y*_ is chosen to contain all the intensity of the diffraction spot. The integral over *q*_*z*_ is understood as the area of a Gaussian (or Lorentzian if that is the observed shape) fitted to this intensity as a function of *q*_*z*_. This is analogous to using the area under a rocking curve in the quasi-monochromatic SANS case to establish the integrated intensity.

## 4. Instrument setup

Our neutron measurements were carried out on the High Magnetic Field Facility for Neutron Scattering (Prokhnenko *et al.*, 2017 ▸). This facility is no longer operational, but at the time of our experiments it consisted of the High Field Magnet (HFM) (Smeibidl *et al.*, 2016 ▸) and the Extreme Environment Diffractometer (EXED) (Prokhnenko *et al.*, 2015 ▸) at the Helmholtz-Zentrum Berlin (HZB). The multi-purpose EXED instrument operated in TOF mode, with a wide range of incident neutron wavelengths, maximizing the volume of reciprocal space that could be observed for a given orientation of the HFM. This was a hybrid solenoid magnet system with a maximum field of 25.9 T, making it the highest continuous magnetic field that was available in the world for neutron scattering experiments. The instrument was used for a wide variety of elastic diffraction or inelastic scattering experiments; in this case we used a setup appropriate for elastic small-angle scattering from the vortex lattice (VL), which has a repeat period of ∼100 Å under our conditions. The direction of the horizontal magnetic field, and therefore of the sample, was rotated relative to the incoming beam by a few degrees, so as to satisfy the Bragg scattering condition at a wavelength near the middle of the range for the incoming neutrons.

**Figure 4:**
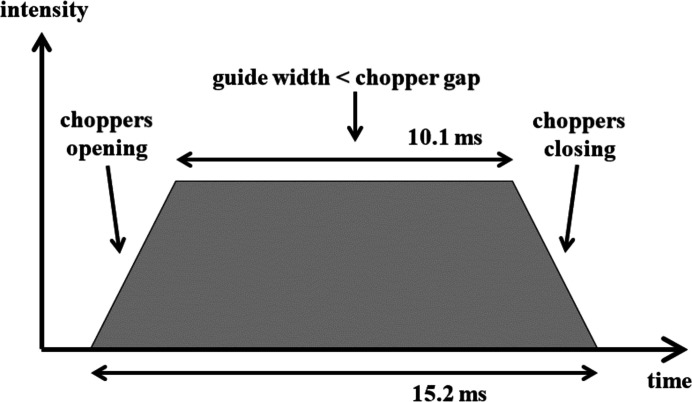
The neutron pulse shape in our experiments. This pulse is repeated every 100 ms.

The neutron beam from the HZB reactor was chopped at a frequency of 10 Hz by two counter-rotating choppers with opening angles of ∼55° situated 53 m before the sample. The gap both opens and closes linearly with time, with a total opening time of 15.2 ms. However, the maximum width of the chopper gap is roughly three times larger than the guide width, so the beam is fully transmitted over the central ∼10.1 ms, giving a flat-topped pulse, as shown in Fig. 4 ▸. We use this pulse shape in our resolution calculations later in the paper, where we represent its width by a single number, the root-mean-square (r.m.s.) time width of the intensity distribution.

Neutrons over the wavelength range 2.5–9.3 Å could be identified by their time of arrival at the detector, between ∼37 and 137 ms after they had passed through the source choppers. Additional choppers removed neutrons which would have arrived at the edges of this time range or outside it. This avoids ‘frame overlap’ with the next pulse arriving 100 ms later (Prokhnenko *et al.*, 2015 ▸). For our first experiment, the data were analysed over the wavelength range 2.55–8.15 Å, ignoring low intensities at the extremes of the range. The incoming beam was collimated by a 30 mm diameter aperture located 5.5 m before the sample and a second 14 mm diameter aperture 1.0 m before the sample, with a sample-to-detector distance of 5.5 m. For the second experiment, the range was 5.0–8.15 Å to reduce background due to double Bragg scattering, first from one twin domain and then from another domain. This is seen only below the Bragg cut-off, *i.e.* at short wavelengths, and from a sample containing both twin domains. The collimation in this case was 30 mm at 7 m with another 12 mm aperture at 0.65 m and a detector distance of 4.5 m. The sample areas were defined by a 7 × 5 mm hole in a cadmium ‘window frame’ around the mosaics. This gave an angular divergence of the incoming beam of ∼0.20° FWHM in the first experiment and ∼0.17° FWHM in the second.

Two different detectors were used in the two experiments; both consisted of arrays of cylindrical position-sensitive detector tubes. The tube spacing defined their horizontal resolution while the vertical resolution was given by 1% of their length. In the first, the detector is that described by Prokhnenko *et al.* (2017 ▸). This detector was at a fixed position with respect to the laboratory and had pixels of size (δ*x*, δ*y*) = (14, 9) mm. In the second case, the detector was attached to the magnet, and so both rotated with the same angle α. This detector also had larger pixels: (20.8, 24) mm. The first detector had better resolution but was not in a vacuum as it was separate from the magnet, so helium or argon gas was put in the flight path to reduce background due to air scattering. Equation (5) gives the relationship between pixel size and *q* resolution.

In both experiments, a perforated cadmium beamstop of measured transmission (∼1.5%) was set to intercept most of the incident beam to avoid overloading the detector. The fraction of the beam transmitted by a perforated beamstop also acts as a beam monitor. This gives a measure of the main beam intensity with the same detection efficiency as the scattered beam and – in TOF mode – with the same time structure. The alternative arrangement of a beam monitor in the incident beam will give a wavelength-dependent efficiency, which in general will be different from that of the detector. In TOF mode, corrections would have to be made for monitor efficiency and the different distance of the monitor from the choppers. In quasi-monochromatic mode, a monitor in the incident beam is not a problem, as long as a perforated attenuator of known transmission is used to measure the main beam intensity. In both cases, one relies on the transmission through the perforations being determined by the fraction of area they occupy, which allows us to make the reasonable assumption that the transmission is wavelength independent.

## 5. Data treatment

The data treatment for obtaining the VL was performed using the software package *Mantid* (Arnold *et al.*, 2014 ▸) using the following procedure.

(i) Before starting the data treatment, we first loaded data files containing information on the position of the direct beam on the detector for each angle of rotation of the magnet at which measurements were taken. These define the centre of our detector, *i.e.* **q** = 0.

(ii) We then loaded separately all of the data files, designated as foreground (being studied to see if there is a VL signal) and background (no VL signal possible). Each file was labelled with an identification number, the angle of rotation of the magnet, and the temperature and magnetic field applied during that measurement. Each foreground file had a corresponding background file measured at the same magnet angle of rotation and both should have been measured at the same field. Background files were obtained by measuring above the critical temperature of the sample. Each file contained the neutron counts collected during a run (usually around 60 min) assembled into 256 logarithmically spaced time bins for each pixel of the detector.

(iii) Next, the data were re-binned from time bins to wavelength bins of width 0.02 Å.

(iv) Each foreground and background file was then divided by the known pixel-dependent efficiency of the detector.

(v) Using the detector centre appropriate for the relevant magnet rotation angle, these files were re-binned from neutron counts versus wavelength in each detector pixel to neutron counts versus wavelength in pixels of the **q** space of the sample (see Section 3).

(vi) Where files had been measured at the same temperature and magnetic field but at different rotation angles, the files were merged at this point and the counts for all angles were added together into the appropriate **q**-space pixel and wavelength bins. It is important to note that *Mantid* conserves the number of neutron counts, adding them to the appropriate bins in the **q**-space pixels.

(vii) A separate workspace was then created to allocate the main-beam wavelength distribution information to every detector pixel. The main-beam information was obtained from the counts passing through the perforated beamstop, corrected by its transmission factor. These data were also rebinned to **q** space in the same manner as the foreground and background files. This process assigned the number of main-beam counts that could have given rise to the scattered intensity in each **q**-space pixel and wavelength bin.

(viii) After this re-binning the separate foreground and background files containing the scattered counts were normalized by the main-beam intensity. The normalized intensities were then summed over wavelength bins as in equation (8) for each **q**-space pixel.

(ix) The normalized background was subtracted from the normalized foreground to give the diffraction signal – from the VL in our case. For our VL data, this was concentrated near the plane perpendicular to the magnetic field (defined as the *q*_*z*_ = 0 plane) in diffraction spots from the VL at **q** = (*q*_*x*_, *q*_*y*_).

(x) We note that determining the integrated intensity from any mesoscopic crystal structure investigated by SANS would require a similar analysis procedure. In the case of a skyrmion lattice, this would give the form factor of the spatially varying magnetization perpendicular to the scattering vector.

**Figure 5:**
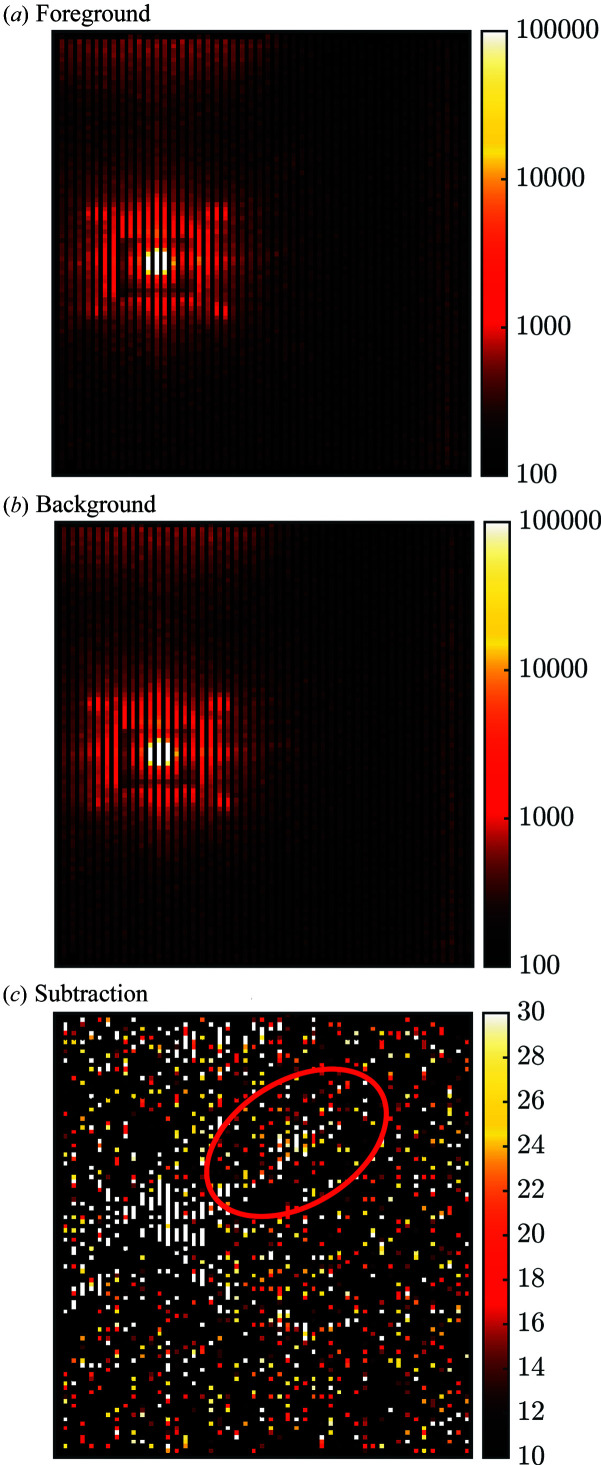
The upper two panels compare the intensity measured on one of the EXED detector panels (there were four detector panels in total, two forward and two backward) after counting foreground and background for 4 h each. The detector pixels are not square. The data in panels (*a*) and (*b*) are displayed using a logarithmic colour scale, and the features seen in both are discussed in the main text. The effect of the perforated beamstop is clear in these panels. Panel (*c*) shows the foreground minus the background, un-smoothed and with a linear colour scale. The double diffraction signals present in the foreground and background cancel, but Poisson errors in the large counts near the main beam are seen in the difference signal. As indicated by the geometry in Fig. 1 ▸, the VL signal is diffracted at different angles depending on wavelength, so is spread out on the detector. For this magnet rotation angle, they appear as faint diagonal streaks to the right of the beam centre; one of them is inside the red ellipse in panel (*c*).

Figs. 5 ▸(*a*) and 5 ▸(*b*) provide examples of foreground and background raw data taken on the 98% detwinned YBa_2_Cu_3_O_7_ sample at 23 T. These display the intensity per pixel of the detector, summed over all neutron wavelengths. The bright spot is the direct beam. Fig. 5 ▸(*c*) shows the corresponding subtraction. In both foreground and background there are four spots of intensity forming a square pattern around the direct beam (note that the detector pixels are not square). These spots are the results of a double diffraction process, firstly from the majority twin and then from the minority twin, or *vice versa*. They are not related to the VL. This occurs at a specific wavelength controlled by the Bragg condition for the crystal planes in question. The actual VL diffraction signal is spread out over a range of distances from the main beam as it appears at all wavelengths, and it is most clearly seen after converting from detector pixels to **q** space. To see the signal clearly in the detector panel output prior to the rest of the analysis required at least 4 h of counting time for both foreground and background.

**Figure 6:**
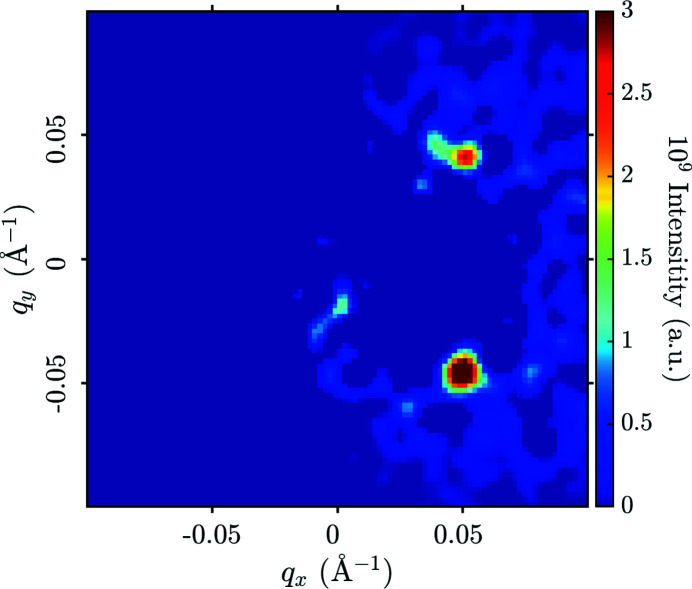
Data from YBa_2_Cu_3_O_7_ at 23 T, measured at α = 1.2 and 2.0°. The colour plot shows the intensity in each pixel after the processing described in the main text, excepting the normalization step. This results in giving the intensity near *q*_*z*_ = 0. The image has been smoothed using *Mantid*.

**Figure 7:**
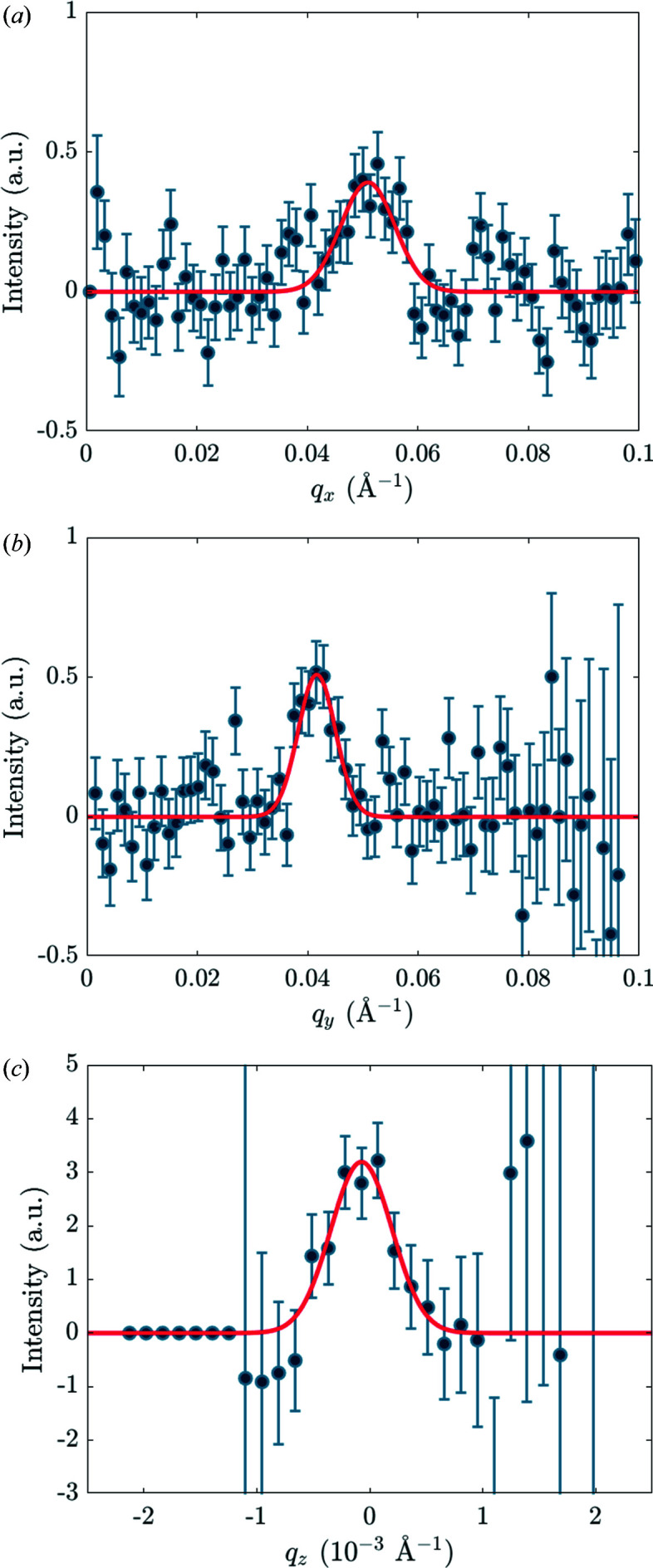
Plots of the VL signal versus *q*_*x*_, *q*_*y*_ and *q*_*z*_ for the upper VL Bragg reflection from YBa_2_Cu_3_O_7_ at 23 T (see Fig. 5 ▸). The signals plotted have been summed over suitable ranges of the other two dimensions of **q** space. The *q*_*z*_ integration gives the final integrated intensity to be carried forward. Normalization by the main beam sometimes results in large uncertainties or even divide-by-zero errors in regions near to or outside the edge of the region covered.

To extract the integrated intensity of the Bragg reflection, a sum over *q*_*x*_, *q*_*y*_ and an integration over *q*_*z*_ must be carried out, as described in the text following equation (8). Fig. 6 ▸ shows an example of the data prior to this integration. Firstly, the signal versus *q*_*x*_ and then versus *q*_*y*_ is plotted in Fig. 7 ▸. In each case, the quantity plotted has been summed over the spot area in the other two dimensions of **q** space. This gives the position and width of the spot, which determine the area of *q*_*x*_ and *q*_*y*_ over which to sum the signal. Finally the *q*_*z*_ integration is done to give the total integrated intensity. As shown in Fig. 7 ▸, all of the fits were Gaussians with the background level constrained to be zero. From the fits, the FWHM in a given direction can also be extracted; this is discussed in more detail in the following section. The dimensions of the *q*_*x*_ *q*_*y*_ area used to sum the signal shown in Fig. 7 ▸(*c*) were set to be ±3× the Gaussian widths of Figs. 7 ▸(*a*) and 7 ▸(*b*).

We have checked our analysis by comparison with quasi-monochromatic measurements and find excellent quantitative agreement in the measured form factor for YBa_2_Cu_3_O_7_ as a function of field (Campillo, Bartkowiak *et al.*, 2022 ▸) when using the first detector setup. For the detector setup in the second experiment, we consistently recorded an integrated intensity lower than that obtained in the first experiment or quasi-monochromatic measurements on three different samples. The measured form factor from the second setup had to be multiplied by 1.5 to match the values measured by other setups. We do not know the exact reason for this but the most likely cause would be a problem with correctly establishing the main beam intensity.

## 6. General considerations

In a TOF experiment, there is often a choice of the range of wavelengths to be used, which will give rise to a range of *q*_*z*_ at the diffraction spot. However, a wide range may not give much extra information, because the intensity of the beam will fall off at long wavelengths and the reflectivity of the sample falls off at short wavelengths as λ^2^. Also, short wavelengths may diffract from the main crystal structure as well as from the long-period structure to be examined. This can give unwanted background, as observed in YBa_2_Cu_3_O_7_ near the main beam in Fig. 5 ▸. This was much more important in our Y_0.85_Ca_0.15_Ba_2_Cu_3_O_7_ sample, because it had equal populations of both twin domains, giving much stronger double Bragg scattering. In this case, as described in Section 4, the short-wavelength data had to be removed from the analysis to reduce the background.

**Figure 8:**
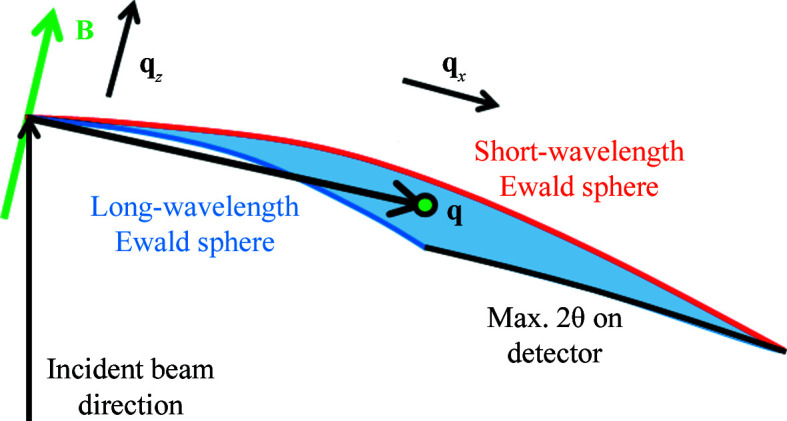
Illustration of the area of **q** space covered by a given range of wavelengths and 2θ values (angles exaggerated for clarity). The directions of *q*_*x*_ and *q*_*z*_ are marked. The magnet is rotated to bring the desired **q** vector into the middle of the shaded area, but for a given rotation angle the area is narrow and curved so that it covers only part of the *q*_*z*_ = 0 plane which contains **q**, and a very short range of *q*_*z*_.

**Figure 9:**
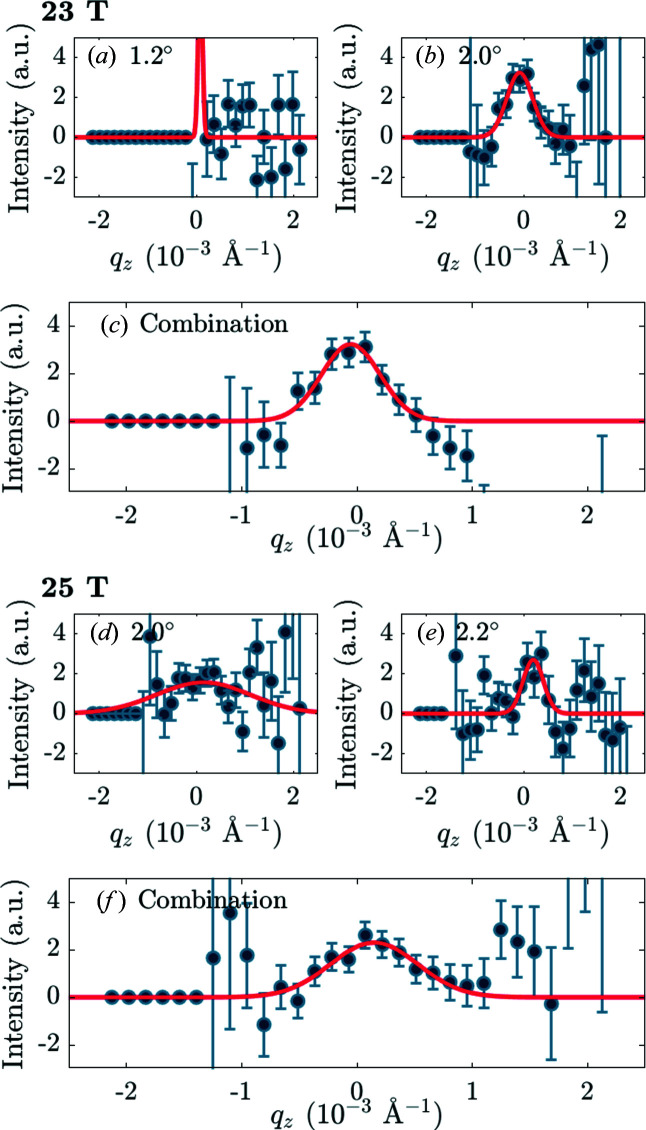
Plots of the VL signal versus *q*_*z*_, (*a*), (*b*) and (*c*) for the upper VL Bragg reflection from YBa_2_Cu_3_O_7_ at 23 T (see Fig. 5 ▸) and (*d*), (*e*) and (*f*) for the lower VL Bragg reflection from YBa_2_Cu_3_O_7_ at 25 T. In panels (*a*), (*b*), (*d*) and (*e*) the magnet angle α is written in the figure. (*c*), (*f*) Data after combination of the two angles shown in panels (*a*) and (*b*), and (*d*) and (*e*), respectively. From the 23 T panels, we can see that the 1.2° data [panel (*a*)] make no contribution to the final combination in panel (*c*) and only add noise. From the 25 T panels, we can see that the noise is heavily reduced when combining both angles in panel (*f*). Note that some data points appear to have zero error: this is because the data are zero.

The rotation angle of the sample and the range of wavelengths determine the region of **q** space covered. A slice in the *q*_*x*_ *q*_*z*_ plane is represented in Fig. 8 ▸. The shape of this region means that some parts of **q** space have little or no scattered signal and also little or no main beam to serve for normalization. If the data are normalized by the main beam, this results in large uncertainties or even divide-by-zero errors in regions near to or outside the edge of the region covered. These effects can be seen in the plots in Fig. 7 ▸, particularly in Fig. 7 ▸(*c*). Taking data at other magnet angles will increase the volume of **q** space that is covered but does not necessarily increase the quantity of useful data about the diffraction spot itself. This is illustrated in Fig. 9 ▸. A good way of dealing with this problem would be to use a Bayesian method of analysis (Holmes, 2014 ▸) which gives little weight to data that have large fractional uncertainties.

Another consideration in SANS measurements is the *q*_*z*_ width of the diffraction spot in the TOF method – or equivalently, the width of the rocking curve in quasi-monochromatic SANS. In the latter case, the width for a new and uninvestigated sample is immediately revealed by a rocking scan. In the TOF case, the range of wavelengths may be too large or too small to obtain efficiently the required data at a single magnet rotation angle. Consider first the situation with a small *q* and a short *z*-axis correlation length, *i.e.* a large *q*_*z*_ width of the signal. Because of the small *q*, the range of *q*_*z*_ covered by the range of wavelengths will also be small (see Fig. 1 ▸) and a set of different sample rotation angles will be required to give sufficient *q*_*z*_ coverage. Conversely, if the sample exhibits a well ordered structure at a large value of *q*, the ‘rocking curve’ given by the TOF technique may be too wide, with many of the data giving background instead of signal. The most challenging situation for SANS by any technique is a weak signal at low *q* with large *q*_*z*_ width, where efficiently setting the required measurement conditions is somewhat more involved for the TOF technique.

## 7. Instrument resolution and VL perfection

**Figure 10:**
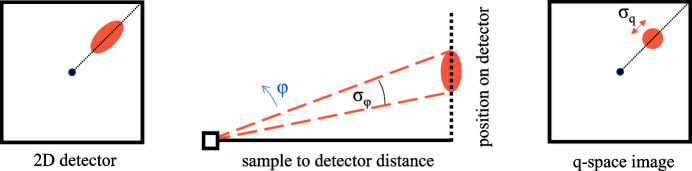
(Left) A schematic illustration of a Bragg spot on a 2D detector with some radial width, corresponding to (centre and right) a wavevector spread of σ_*q*_. This can also be represented by an angular width σ_φ_ of the beam coming from the sample to give rise to that Bragg spot. This angular width is determined by the incoming beam collimation, the mosaic spread of the VL and the spread of Bragg angles due to imperfect monochromation of the beam. The diffraction angles in this image are exaggerated for clarity.

In this section, we discuss the questions of instrument resolution and measurement of the perfection of the structure being investigated within the context of VL diffraction. However, the results can equally be applied to other mesoscopic structures to relate the coherence of these structures in the *x*, *y* and *z* directions to the spot widths in **q** space. The width of a diffraction spot in *q*_*x*_, *q*_*y*_ and *q*_*z*_ will be determined partly by the instrument resolution (which is highest in the *z* direction) and partly by the perfection of the VL. A simple approach in the quasi-monochromatic case is described by Cubitt *et al.* (1992 ▸).^2^ This analysis was carried out not in **q** space but in terms of the widths of distributions over angles, including any spread in the Bragg angle. It showed how the effects of the angular spread and wavelength spread of the incoming beam combine with any imperfection of the VL to give the widths of the diffraction spot on the detector and of its rocking curve. In equation (3) of Cubitt *et al.* (1992 ▸), the intensity of a diffraction spot is given as a function of two variables, both treated as pointing radially from the main beam through the diffraction spot: (i) the angle φ of the scattered beam measured relative to the ideal Bragg 2θ_B_ of the spot, and (ii) the rocking angle ω measured relative to the ideal Bragg θ_B_. We extend slightly the treatment of Cubitt *et al.* (1992 ▸) to derive the instrument *q*_*z*_ resolution, which was not explicitly derived there, and to calculate the *q*_*x*_ *q*_*y*_ resolution for the TOF case. In a quasi-monochromatic SANS experiment, the contributions of the instrument to the resolution are given by the instrument setup, and the contributions of the sample may be extracted from the data as angular widths. The outputs obtained from the analysis of TOF data are in **q** space, and so to use the results of Cubitt *et al.* (1992 ▸) it is convenient to convert values from TOF observations into angles. The radial width σ_φ_ of a spot in the plane of the detector is related to the observed radial width σ_*q*_ by σ_φ_ = σ_*q*_/*k*_*j*_ (see Fig. 10 ▸). However, because of the range of wavelengths used in TOF, we can only apply this expression approximately, using a value in the middle of the wavelength range. The equivalent of the rocking curve width σ_ω_ is given in terms of the observed width 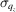 by 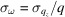 . Note that this implies that, for a rocking curve measured by quasi-monochromatic SANS to have an angular width which is independent of field, this would correspond to a *q*_*z*_ width which increases with *q* and therefore with field.

Cubitt *et al.* (1992 ▸) used the following symbols: *a* is the angular width of the incoming collimation, *b* is the mosaic spread of the VL crystal and *c* is the spread of Bragg angles. To calculate the rocking curve width for a spot, we have to integrate equation (3) of Cubitt *et al.* (1992 ▸) over φ to obtain the total intensity of the spot as a function of ω. This was not done explicitly by Cubitt *et al.* The result is

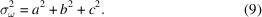

This simple addition of the three widths applies in both quasi-monochromatic and TOF setups. However, for the spot size different expressions may need to be used. If in a quasi-monochromatic experiment the spot width is measured at fixed ω = 0, the following expression applies: 

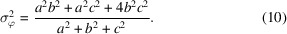

However, in a TOF experiment, we are effectively sampling a range of ω values so we have to integrate equation (3) of Cubitt *et al.* (1992 ▸) over ω, obtaining the much simpler expression

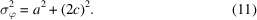

This result [not given by Cubitt *et al.* (1992 ▸)] may also be used in the quasi-monochromatic case if the spot width is measured on a sum over a rocking curve. We note that equation (11) shows that the spot size does not depend on the mosaic spread *b* of the sample. In the quasi-monochromatic case, the ω = 0 spot size in equation (10) does give information about *b*. Furthermore, as discussed by Cubitt *et al.* (1992 ▸), a different expression containing *a*, *b* and *c* may be obtained from the slight shift in spot position as a function of rocking angle ω. To obtain similar information from TOF data, one would have to analyse thin slices of the wavelength distribution to obtain quasi-monochromatic data.

To use these expressions we must calculate the parameters. First we consider the spread of Bragg angles, which can arise either from wavelength spread or from variation in the *d* spacing of the VL or other mesoscopic crystal. For a VL, it is usually the case that the magnetic field is extremely uniform, so *c* tends to be dominated by the wavelength spread. The data from a TOF instrument will be collected into bins with arrival time *t* for each pixel of the detector, but if these bins are chosen correctly they will not determine the wavelength resolution of the instrument. This is instead controlled by the pulse width of the incoming beam, often set by input chopper(s).

In our case, the pulse shape shown in Fig. 4 ▸ gives a 2 × r.m.s. width of δ*t* = 7.3 ms. The wavelength resolution arising from the width of the incoming neutron pulse is given by Δλ/λ = Δ*t*/*t*, and in our case varied from 2 × r.m.s. ∼22% at 2.55 Å to ∼7% at 8.15 Å. This may also be expressed by stating that Δλ ∝ Δ*t* is independent of λ and in our experiment was equal to ∼0.55 Å. Pautrat *et al.* (2012 ▸) reduced Δ*t* using choppers to improve the wavelength resolution in their TOF experiment on the VL in niobium. This has a side effect of reducing the flux.

The other contributions to the resolution of the instrument are the input collimation and the detector pixel size. In the first experiment setup, the ∼0.20° input-beam divergence projected to a ∼19 mm FWHM spot size on the detector at 5.5 m, which had a pixel size (δ*x*, δ*y*) = (14, 9) mm. For the second setup, with a detector less suitable for SANS located 4.5 m from the sample, the calculated spot size was ∼13 mm FWHM, compared with (δ*x*, δ*y*) = (20.8, 24) mm. Thus in the first case the resolution in the detector plane was limited by input collimation, and in the second by pixel size.

**Figure 11:**
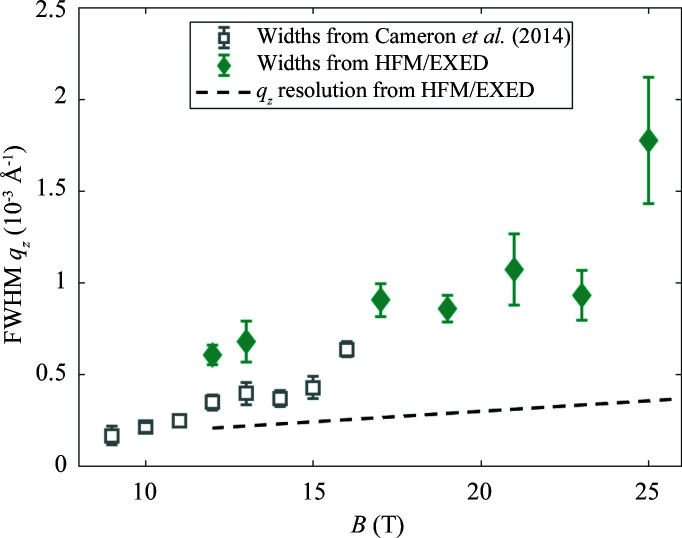
The *q*_*z*_ FWHM widths of the diffraction spots at base temperature versus field above 8 T. The dashed line is the calculated *q*_*z*_ resolution. The empty square points are from a previous non-TOF study (Cameron *et al.*, 2014 ▸).

We calculate only for the first setup, relating to the data shown in Figs. 11 ▸ and 12 ▸, and demonstrate the method for *B* = 20 T and a wavelength of 6 Å in the middle of the TOF range. We have *a* = 0.20° and calculate *c* = 0.16° from the instrumental wavelength spread. (The spread of the Bragg angle δθ_B_ ≃ δλ/2*d*, with δλ ≃ 0.55 Å and *d* ≃ 100 Å at 20 T.) Using equation (9) we calculate the FWHM of σ_ω_ = 0.27° due to just these two terms. However, with 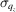 ≃ 10^−3^ Å^−1^ from Fig. 11 ▸, we calculate (using 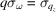 ) a much larger σ_ω_ ≃ 0.9°. This indicates that, in the *q*_*z*_ direction, the VL mosaic *b* (or alternatively the *z* correlation length of the VL) dominates the observed *q*_*z*_ width, which is much larger than the instrument resolution due to input collimation and wavelength spread denoted by *a* and *c*.

**Figure 12:**
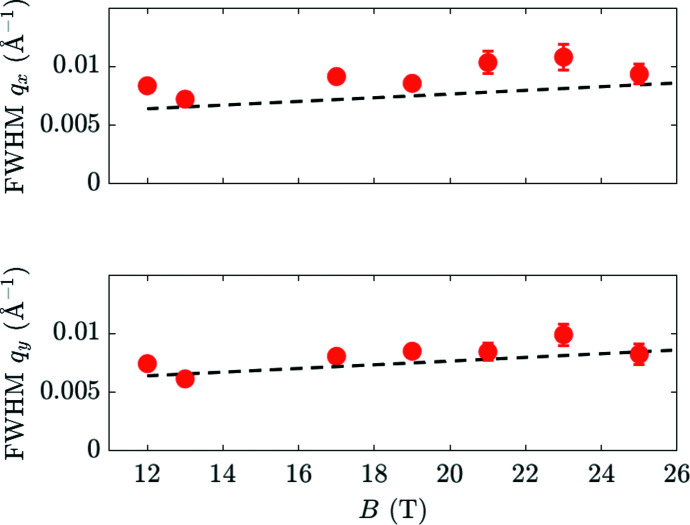
The FWHM widths at 3 K of the diffraction spots in the *q*_*x*_ and *q*_*y*_ directions versus field. These widths are mainly dominated by the in-plane resolution of the instrument – marked by dashed lines – so they only set a low limit on the VL perfection. Dashed lines indicate the resolution of the HFM/EXED instrument.

Now we turn to the size of the spots in the *q*_*x*_ *q*_*y*_ plane. At 20 T, equation (11) gives σ_φ_ ≃ 0.42°, and hence σ_*q*_ ≃ 8 × 10^−3^ Å^−1^. The calculated radial *q* resolution is comparable to the *q*_*x*_, *q*_*y*_ widths observed in Fig. 12 ▸, so the circular spot that we see is resolution limited. This only confirms that the spread of Bragg angles *c*, which is the only quantity in equation (11) possibly depending on VL properties, is dominated by wavelength resolution and cannot give information about any spread in the VL *d* spacing.

## 8. Summary

We have shown how to extract quantitative information from VLs and other long-period structures, such as skyrmions, using a TOF instrument. The range of wavelengths is equivalent to sampling a range of rocking angles at once. However, sometimes using a large range of wavelengths can give additional background. The choice of magnet angles at which to collect data is very important and is challenging to determine in advance of the measurement, especially if the VL structure or sample quality is not already known. The strategies presented here will be useful for the high-flux TOF SANS instruments at pulsed sources.

## Footnotes

2 There is a misprint in equation (2) of Cubitt *et al.* (1992 ▸), where integration over ω should be over ν, but the following results are correct.
